# Correction: Pauzaite et al. Dbf4-Cdc7 (DDK) Inhibitor PHA-767491 Displays Potent Anti-Proliferative Effects via Crosstalk with the CDK2-RB-E2F Pathway. *Biomedicines* 2022, *10*, 2012

**DOI:** 10.3390/biomedicines14081830

**Published:** 2026-08-14

**Authors:** Tekle Pauzaite, James Tollitt, Betul Sopaci, Louise Caprani, Olivia Iwanowytsch, Urvi Thacker, John G. Hardy, Sarah L. Allinson, Nikki A. Copeland

**Affiliations:** 1 Biomedical and Life Sciences, Faculty of Health and Medicine, Lancaster University, Lancaster LA1 4YQ, UK; tp471@cam.ac.uk (T.P.); j.tollitt@lancaster.ac.uk (J.T.); b.sopaci@lancaster.ac.uk (B.S.); l.caprani@lancaster.ac.uk (L.C.); o.iwanowytsch@lancaster.ac.uk (O.I.); u.thacker@lancaster.ac.uk (U.T.); s.allinson@lancaster.ac.uk (S.L.A.); 2 Materials Science Institute, Lancaster University, Lancaster LA1 4YW, UK; j.g.hardy@lancaster.ac.uk; 3 Department of Chemistry, Faculty of Science and Technology, Lancaster University, Lancaster LA1 4YB, UK

## Error in Figure

In the original publication [1], there were duplicated blots in Figure 2A,C. These errors arose during publication/revision in the cropping and display of blots for MCM2 pS40/41, MCM2pS53 and Rb leading to duplication of the blots. This was not detected during the publication process. These errors were corrected using western blots from the original dataset. The full blots and cropping can be seen in the Supplementary Materials. The errors from the original paper but have been corrected below. These errors do not affect the original interpretation and the remainder of the paper remains unchanged.

**Figure 2:**
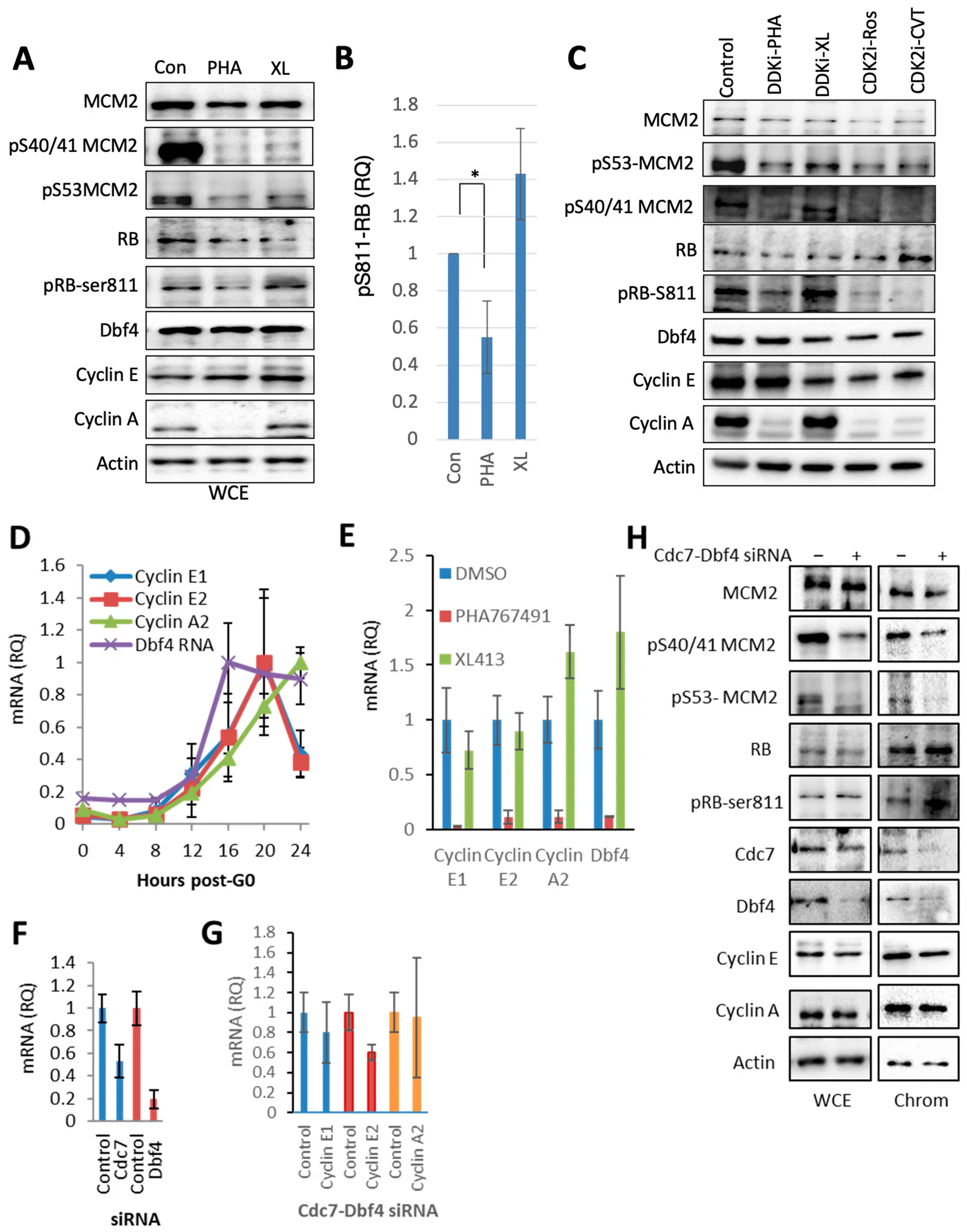
Off-target PHA-767491 activity inhibits E2F-mediated transcription of the cell cycle regulators cyclin E1, cyclin E2, Dbf4 and cyclin A2. (**A**) Immunoblot showing the levels of total MCM2, pS53-MCM2 and pS40/41-MCM2 as DDK-specific phospho-sites, cyclin E, cyclin A, Dbf4, RB, RB pS811 as CDK2 phospho-site and actin after DDK inhibitor treatment as indicated. (**B**) Quantitation of RB pS811 relative to RB total showing mean ± standard deviation, *n* = 3, *p* < 0.05 (*) was determined using unpaired 2 tailed t-test. (**C**) As for A, including CDK2 inhibitors roscovitine and CVT-313. (**D**) NIH3T3 cells were synchronised in G0 and stimulated to re-enter the cell cycle. RNA samples were removed at indicated time points and transcript levels were determined by qRT-PCR. Values shown are standardised relative to maximum levels for each gene, showing mean ± standard deviation, *n* = 3. (**E**) Synchronous population was treated with DDK inhibitors 12–20 h after release from G0. The transcript levels of cyclin A2, cyclin E1, cyclin E2 and Dbf4 were monitored relative to GAPDH mRNA levels relative to the levels of the solvent control (DMSO) for PHA-767491 and XL-413 treated cells. Showing mean ± standard deviation, where *n* = 3. (**F**) 3T3 cells were synchronised and siRNA-mediated depletion of Cdc7 and Dbf4 was performed upon release from G0 phase and total RNA extracted 20 h after transfection. Transcript levels for Dbf4 and Cdc7 showing mean value ± standard deviation, which are the average of four independent experiments, each performed in triplicate. (**G**) Quantitation of E2F-regulated transcripts cyclin A2, cyclin E1 and cyclin E2 after CDC7-Dbf4 siRNA treatment from Figure 2F, showing mean ± standard deviation, *n* = 3 independent experiments of with three technical replicates. (**H**) Immunoblot of WCE and chromatin fractions of siRNA transfected cells showing Cdc7, Dbf4, MCM2 and MCM2-pSer40/41, MCM2-pSer53, cyclin E, cyclin A, Dbf4, RB, RB-pS811 and actin.

The authors state that the scientific conclusions are unaffected. This correction was approved by the Academic Editor. The original publication has also been updated.

## Supplementary Materials

The following supporting information can be downloaded at https://www.mdpi.com/article/10.3390/biomedicines14081830/s1.
