# Supplementary material for: Correction: Pauzaite et al. Dbf4-Cdc7 (DDK) Inhibitor PHA-767491 Displays Potent Anti-Proliferative Effects via Crosstalk with the CDK2-RB-E2F Pathway. Biomedicines 2022, 10, 2012
Source: Biomedicines. 2026 Aug 14;14(8):1830. doi: 10.3390/biomedicines14081830 (PMC13509877; doi:10.3390/biomedicines14081830)
Supplement: Supplementary file 1 [file biomedicines-14-01830-s001.zip › biomedicines-4150332-supplementary.pdf]

Figure 2A. Western blots

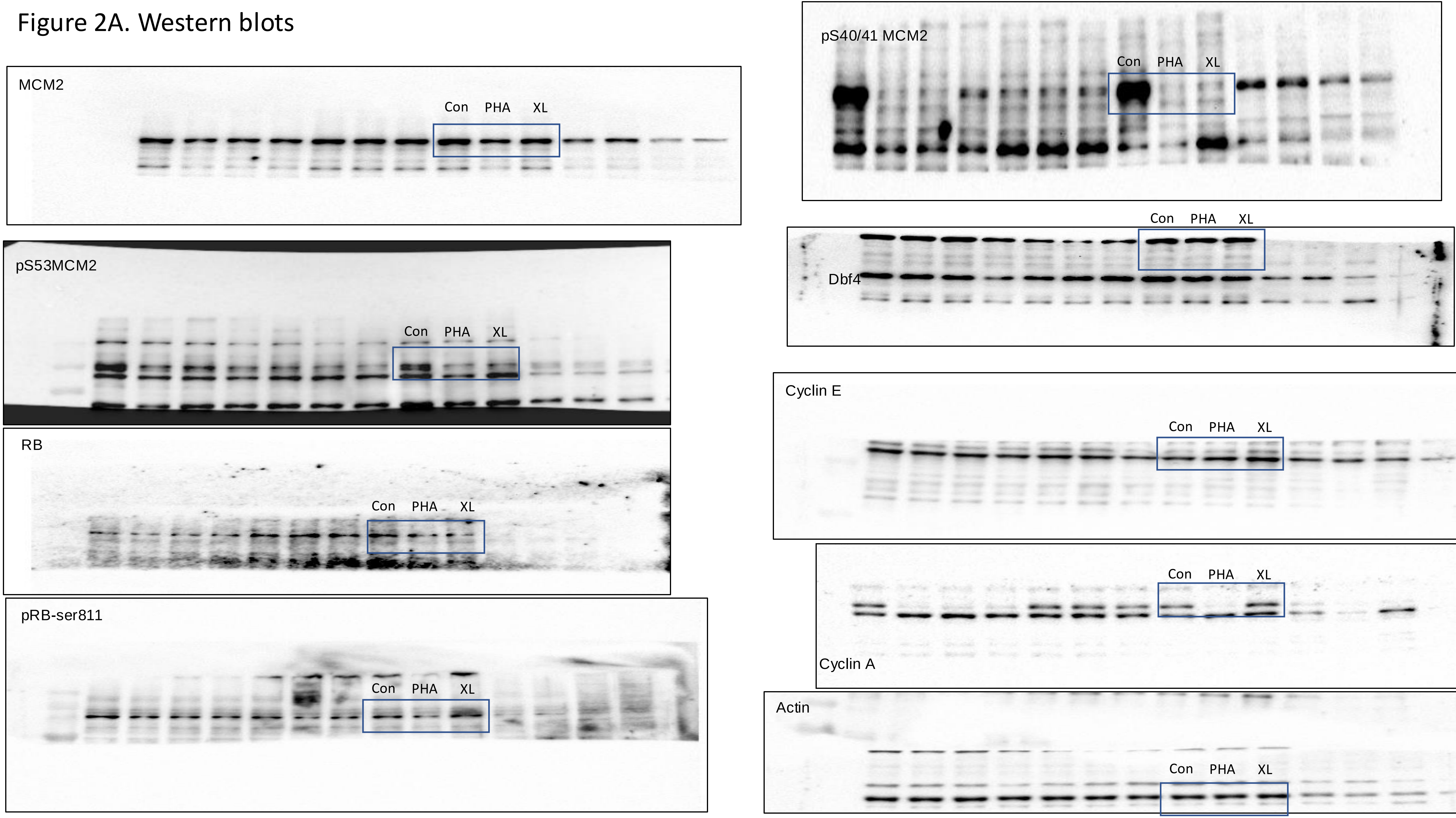

Figure 2C. Western blots

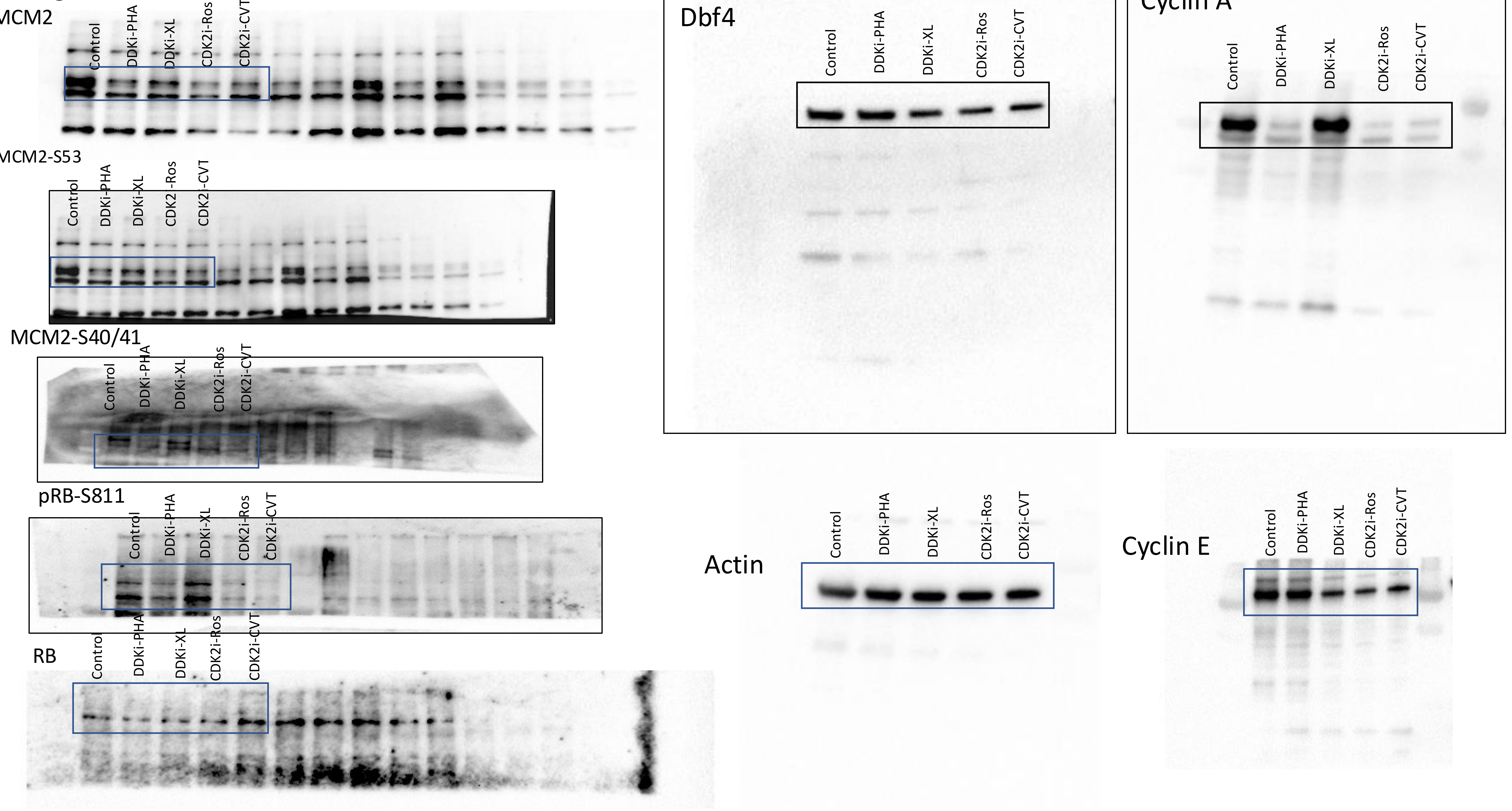

Figure 2G. Western blots

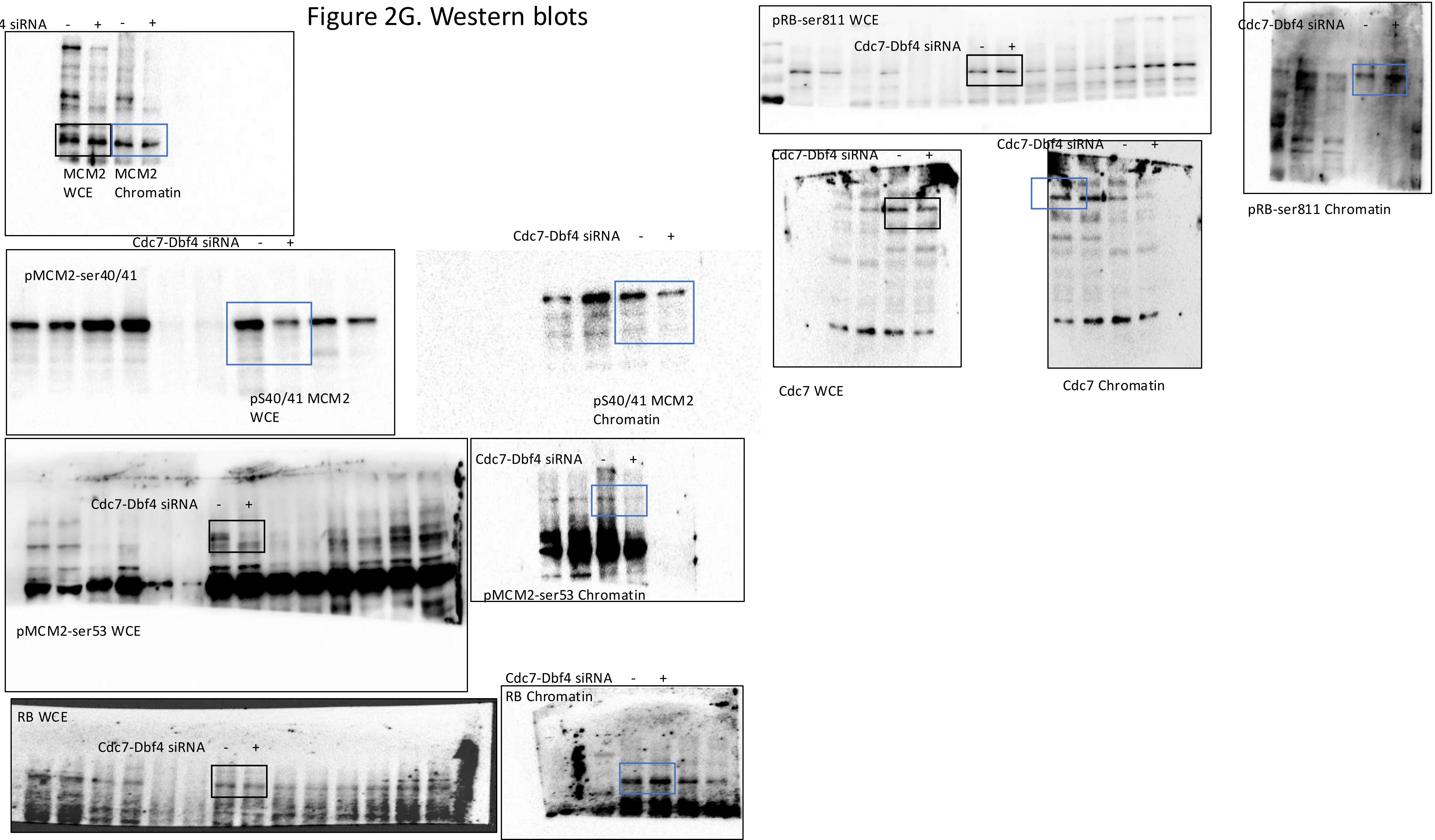

Figure 2G. Western blots cont.

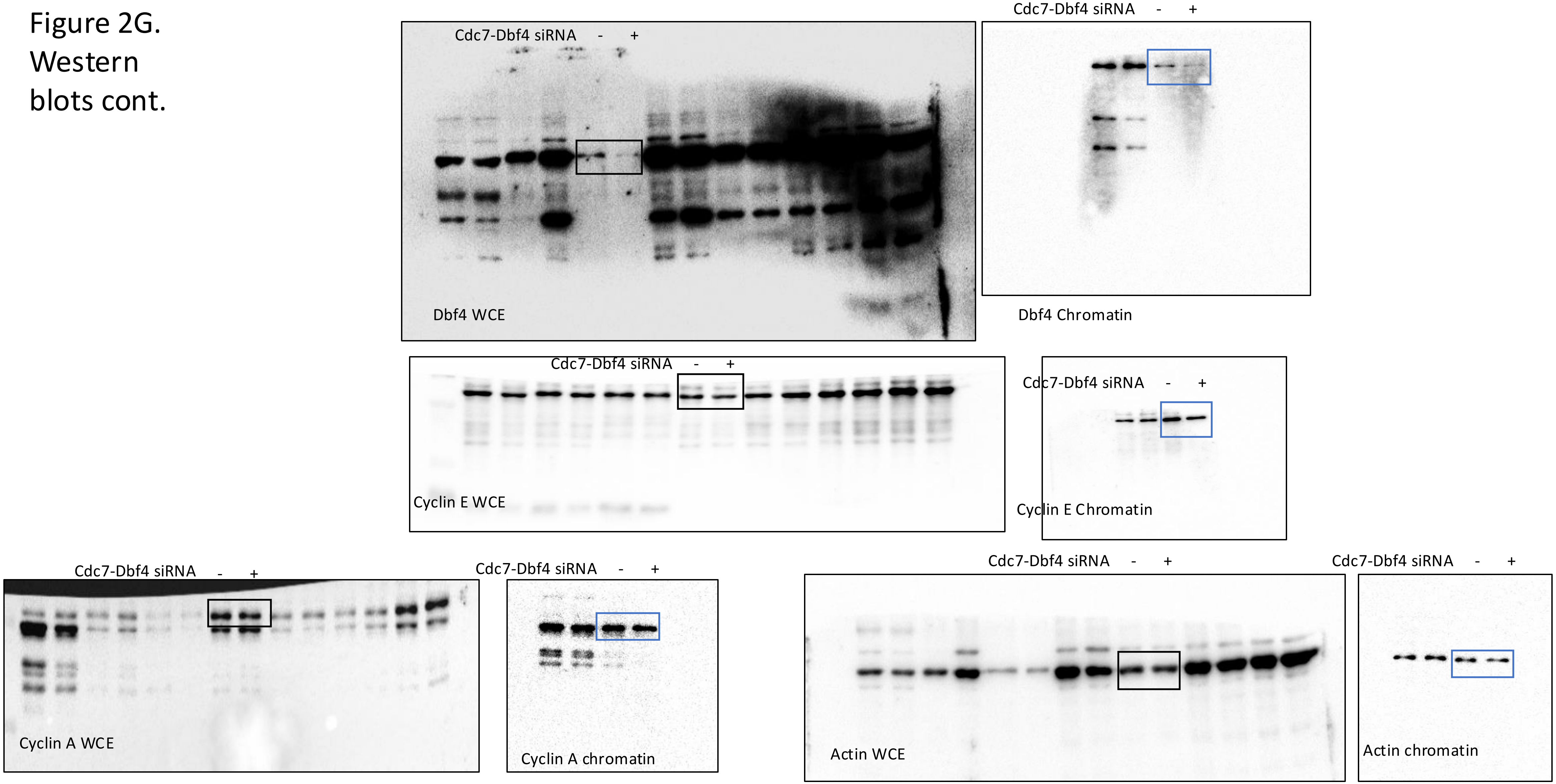

Figure 5D

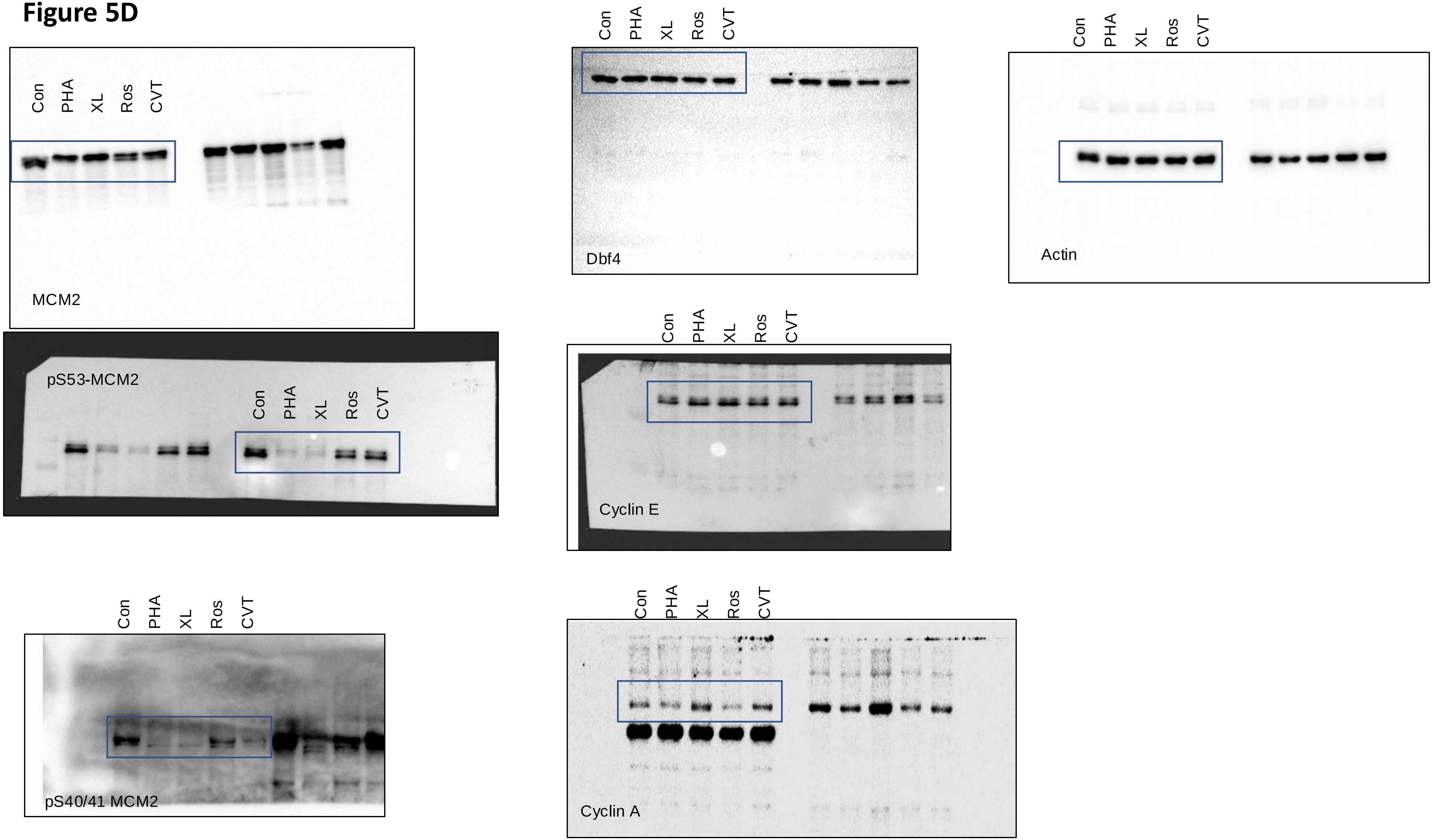

Con
PHA
XL
Ros
CVT
MCM2
pS53-MCM2
Dbf4
Cyclin E
Actin
pS40/41 MCM2
Cyclin A

Figure 5E

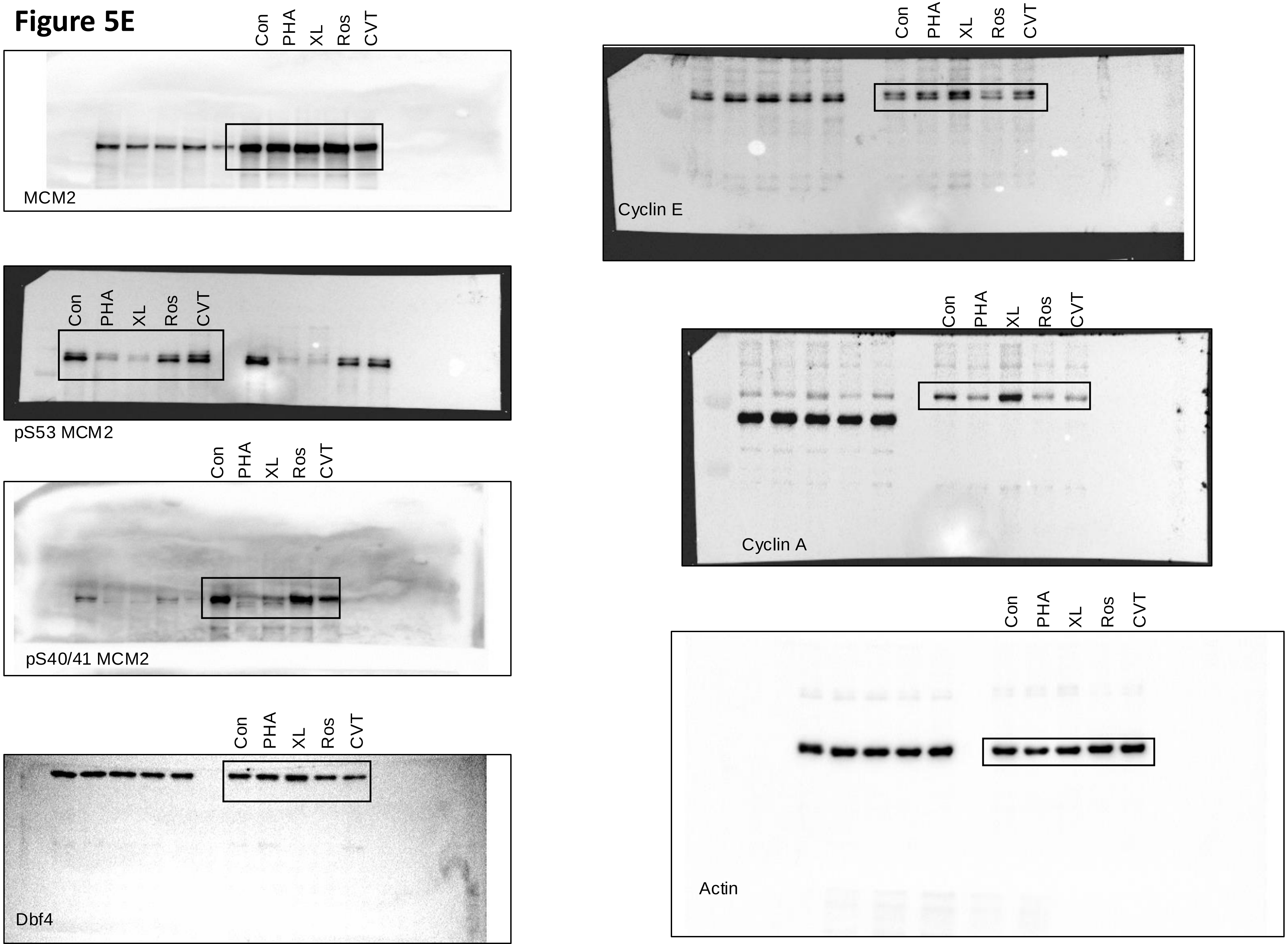
Con
PHA
XL
Ros
CVT
MCM2
pS53 MCM2
pS40/41 MCM2
Dbf4
Cyclin E
Cyclin A
Actin

Figure 5F

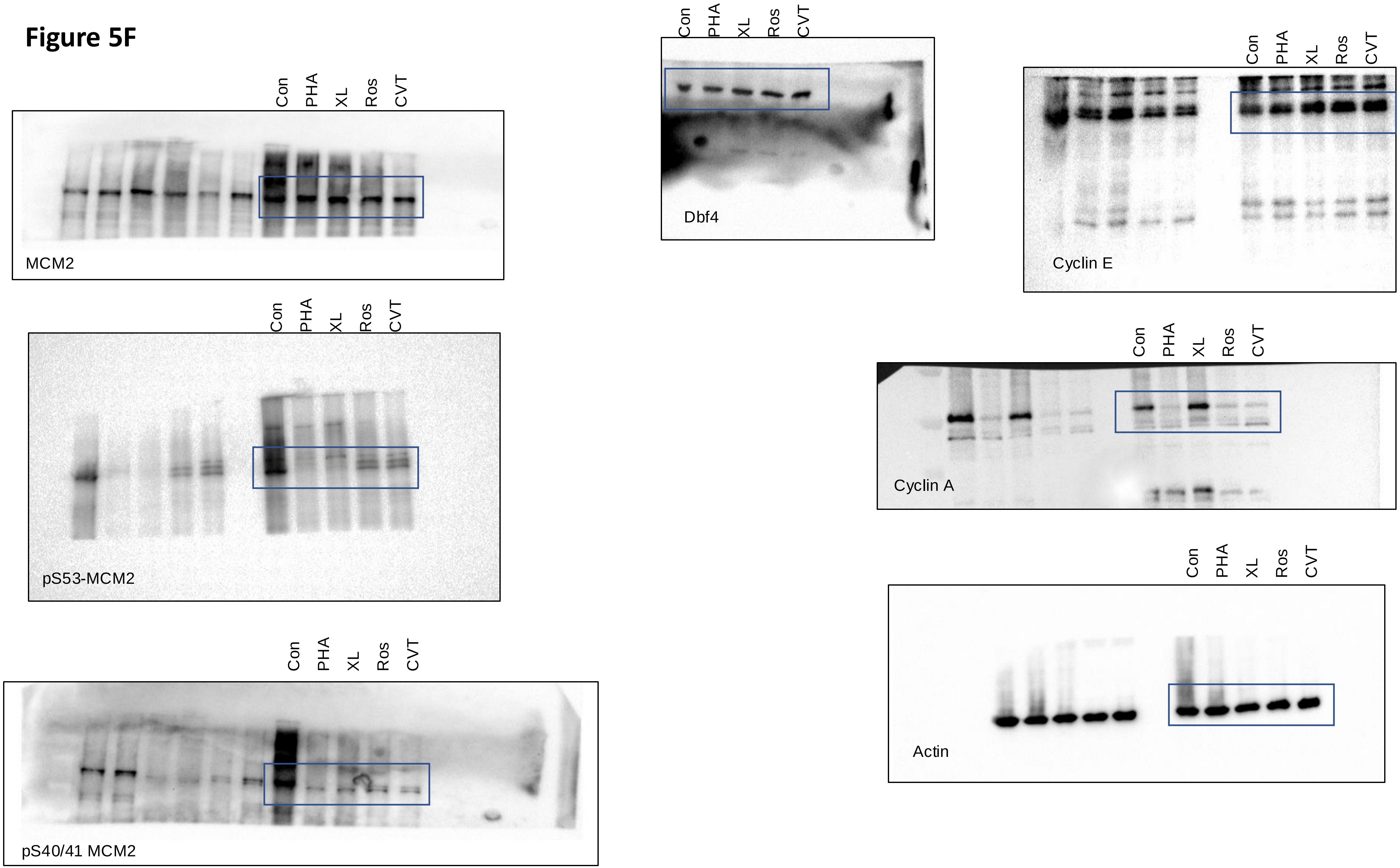

Con
PHA
XL
Ros
CVT
MCM2
pS53-MCM2
pS40/41 MCM2
Dbf4
Cyclin E
Cyclin A
Actin
